# Convolutional neural networks for vision neuroscience: significance, developments, and outstanding issues

**DOI:** 10.3389/fncom.2023.1153572

**Published:** 2023-07-06

**Authors:** Alessia Celeghin, Alessio Borriero, Davide Orsenigo, Matteo Diano, Carlos Andrés Méndez Guerrero, Alan Perotti, Giovanni Petri, Marco Tamietto

**Affiliations:** ^1^Department of Psychology, University of Torino, Turin, Italy; ^2^Institut des Sciences Cognitives Marc Jeannerod, CNRS, Université de Lyon, Lyon, France; ^3^CENTAI Institute, Turin, Italy; ^4^Department of Medical and Clinical Psychology, and CoRPS–Center of Research on Psychology in Somatic Diseases–Tilburg University, Tilburg, Netherlands

**Keywords:** Convolutional Neural Networks (CNN), visual system, ventral stream, blindsight, superior colliculus, pulvinar, V1-independent vision

## Abstract

Convolutional Neural Networks (CNN) are a class of machine learning models predominately used in computer vision tasks and can achieve human-like performance through learning from experience. Their striking similarities to the structural and functional principles of the primate visual system allow for comparisons between these artificial networks and their biological counterparts, enabling exploration of how visual functions and neural representations may emerge in the real brain from a limited set of computational principles. After considering the basic features of CNNs, we discuss the opportunities and challenges of endorsing CNNs as *in silico* models of the primate visual system. Specifically, we highlight several emerging notions about the anatomical and physiological properties of the visual system that still need to be systematically integrated into current CNN models. These tenets include the implementation of parallel processing pathways from the early stages of retinal input and the reconsideration of several assumptions concerning the serial progression of information flow. We suggest design choices and architectural constraints that could facilitate a closer alignment with biology provide causal evidence of the predictive link between the artificial and biological visual systems. Adopting this principled perspective could potentially lead to new research questions and applications of CNNs beyond modeling object recognition.

## The place of Convolutional Neural Networks between neuroscience and cognitive sciences

The brain processes multidimensional and context-dependent information about the world to generate appropriate behaviors. Models in cognitive sciences capture principles of brain information processing but typically overlook fine details about the spatiotemporal implementation of neuronal functions or biological components. On the other hand, neurobiological models recapitulate dynamics of action potentials or signal propagation across neuronal populations. However, they have limited success in understanding the computations that support complex behaviors in real-life contexts (Kriegeskorte and Golan, 2019).

In parallel to research in neuroscience, CNNs have become a powerful tool in machine learning and AI that can attain human-like performance. In fact, CNNs can approximate functions in complex and real-world tasks, such as visual recognition (Krizhevsky et al., 2012), language processing (Wolf et al., 2020), or motor learning (Mnih et al., 2015; Dhawale et al., 2017), in ways resembling biological agents. Fueled by the current success of AI and computer vision, current studies suggest that CNNs can potentially bridge the gap between “disembodied” descriptions of cognitive functions and neurobiological models, thus offering a new framework for predicting brain information processing (Kriegeskorte and Golan, 2019). At its core, this framework explains sensory, cognitive, and motor functions in terms of local computations that emerge from experience in networks of units aggregated in multiple (i.e., deep) layers.

Nevertheless, the synergy between neuroscience and AI remains elusive unless the opportunities and challenges of reframing classic questions in neuroscience as deep learning problems are considered (Saxe et al., 2020). Why should we study biological brains through the lens of CNNs? Which new research question do CNNs allow to emerge in neuroscience? What do they offer more than, or differently from, traditional models in terms of predictions, interpretability, or explanatory power? In this short review, we summarize basic concepts and describe recent progress at the intersection between neuroscience and AI, limiting our discussion to the primate visual system and its functions. We then outline some principles in visual neuroscience that still need to be systematically integrated into current CNN models of the primate visual brain. These principles are cornerstones to improve the neurobiological realism of CNNs, and we propose examples of design choices and architectural constraints that may permit a closer match to biology. To this end, we would like to contribute to setting a roadmap for vision neuroscientists interested in drawing on CNNs toolkit.

## Basic principles of CNNs for vision neuroscience

Convolutional Neural Networks are a particular class of artificial neural networks inspired by the architecture and basic functions of biological vision (LeCun et al., 1989; Gu et al., 2018). In contrast to non-convolutional fully connected networks, where each neuron in a layer is connected to all neurons in the previous layer, CNNs employ local connectivity and shared weights through convolutional layers. In general, a CNN consists of many processing units akin to neurons, arranged in interconnected layers typically interpreted as being analogous to brain areas, and with connections defined by weights that mimic the integration and activation properties of synapses. The output of one stage of operations is typically a non-linear combination of the input received and is then passed on to the next layer. This circuit motif is repeated several times and creates a hierarchical organization until the cascade culminates with a discriminative classification or regression generated by the last layers used for readout. A convolutional layer contains many filters with distinct receptive fields that are applied to the input image through a convolution operation, which allows the network to capture spatial and temporal patterns in the input data. The filter’s weights are the learnable parameters of these layers, with the learning process typically managed by standard gradient descent algorithms (LeCun et al., 2015; Yamins and DiCarlo, 2016; Barrett et al., 2019; Kragel et al., 2019; Hasson et al., 2020).

Historically, the development of CNNs was informed by tuning properties of simple and complex neurons in the primary visual cortex (V1), the major cortical target of retinal information, modeled through handcrafted Gabor filters (Fukushima, 1980; Riesenhuber and Poggio, 2000). This bottom-up approach proved effective in modeling V1 properties, but had limited success when extended to higher-order cortical areas along the visual ventral stream (Gallant et al., 1996; Kriegeskorte, 2009). These limitations contributed to shifting the focus toward a goal-driven approach by maximizing, for example, the classification accuracy (Yamins and DiCarlo, 2016). Instead of characterizing the coding properties of individual neurons to enforce model parameters, the workflow of the goal-driven approach reverses the order: first, optimize the CNN to perform an ecologically relevant visual task, then compare artificial networks to real neural data.

Goal-driven CNNs learn to map input patterns (e.g., raw images) to output classifications (e.g., sorting natural images according to categories like faces, objects, and animals). They learn through training, in the form of supervised feedback or reward signals. Throughout this process, the network self-organizes, meaning that computations emerge spontaneously during training and weights change with repeated exposures to labeled or rewarded images (e.g., using the backpropagation algorithm) (LeCun et al., 2015). On the one hand, the classical pattern of interleaving convolutional with pooling layers produces filters with increasing receptive fields as the network layers are traversed forward. On the other hand, the goal-driven learning process does not explicitly enforce any kind of structure in the internal computations. The researcher examines the population-level description of how computations arise organically over the course of network training. The underlying assumption is that hidden layers of a good network model would functionally behave like real neurons in the corresponding neural structures. It has been observed that CNNs commonly learn hierarchies of abstraction, with the first layers acting as Gabor filters while deeper layers become detectors of more complex patterns (Yamins and DiCarlo, 2016). This emergent property as sparked speculations as to whether trained CNNs can functionally behave like biological neurons in the corresponding neural structures (Khaligh-Razavi and Kriegeskorte, 2014; Kuzovkin et al., 2018; Van Dyck et al., 2021).

We argue that the bottom-up and the goal-driven approaches are not mutually exclusive. It is not futile, indeed, to characterize the coding properties of individual neurons, especially at the early stages of visual analyses (i.e., in the retina, subcortical structures receiving direct retinal input, or V1). Design choices that permit a closer match to biology also improve subsequent model fit to neural data or contribute to explaining the diversity of structural and functional properties in the visual brain. For example, when connections between the first layers are constrained with a bottleneck that reduces neurons at the virtual retinal output, consistent with the anatomy of the optic nerve, early layers of the CNN exhibit spontaneously concentric center-surround responses, as in the thalamus, whereas later layers are tuned to orientations, as in V1 (Lindsey et al., 2019). Likewise, the relationship between high selectivity for orientation in simple cells and low selectivity to spatial phase in complex cells of V1 has long been debated, as some mammals lack orientation maps. Different forms of pooling implemented in a Sparse Deep Predictive Coding (SDPC) model account for the emergence of complex cells in V1, both with and without orientation maps (Boutin et al., 2022). Pooling in the feature space is responsible for the formation of orientation maps, whereas pooling in the retinotopic space is related to the emergence of complex cells. Therefore, CNN approaches to this issue suggest that the presence or absence of orientation maps results from diverse strategies employed by different species to achieve invariance in complex natural stimuli.

Convolutional Neural Networks can thus differ considerably in their objective function (the goal of the system), learning rule (how parameters are updated to improve the goal), and circuit architecture (how units are arranged and connected), which are the three central components specified by design (Richards et al., 2019). From an engineering perspective, CNNs are built to solve a task better than prior models, with less computational effort or fewer training examples. Exceeding human performance is desirable, and biological plausibility is not a driving or required factor. Conversely, a closer correspondence with biological brains is paramount from a neuroscientific standpoint. In the latter case, CNNs can be useful if they incorporate elements that parallel the architecture and principles of functioning of the biological visual system. By doing so, CNNs can offer mechanistic hypotheses and enable empirical exploration of how a pattern of behaviors and neural representations may arise in the real brain from a limited set of computational principles.

## CNN as (partial) models of the visual brain: why and how

The visual system is typically represented as a constellation of different but interconnected maps harbored in anatomically distinguishable areas that analyses diverse input features, such as curvature, color, or motion. The division of labor across these areas is classically charted at the cortical level in two “pathways” or “streams,” the dorsal and ventral, originally conceived to progress linearly and hierarchically from a common antecedent in V1 (Ungerleider and Mishkin, 1982; Goodale and Milner, 1992). Along the ventral stream, which courses from V1 to areas downstream up to the temporal pole, retinotopy decreases, receptive fields become progressively larger, and neural responses are increasingly complex and invariant to low-level changes in the input space. This cascade culminates at the apex of the ventral stream with “concept” cells that are tuned to specific (sub)categories, such as (famous) faces, bodies, or places (Quiroga et al., 2005).

The designed architecture of CNNs, as described herein, parallels that of the ventral stream along several dimensions: hierarchical sequence of organized stages, loose correspondence between different layers and visual areas such as V1, V2, V4 and IT, progressive increase of receptive field size and complexity. These features, combined with the evidence that artificial networks trained on an ecologically relevant task attain human-level performance and learn abstraction hierarchies, make CNNs credible candidates for modeling the ventral stream. However, these structural features and the objective function of CNNs are built by the experimenter. Arguably, artificial networks should exhibit additional properties and representations that are not explicitly engineered and that match those found in biological brains.

### Assessing the behavioral correspondence

The equivalence between CNNs and biological brains can be profitably understood in the context of the behavioral outcomes they produce, beyond the overall accuracy in image classification for which the network has been explicitly optimized. CNNs trained for generic object recognition develop representations and categorical similarity that relate closely to human perceptual shape and semantic judgments (Kubilius et al., 2016). CNNs match human and non-human primate error patterns across object categories, viewpoint variations and similarity judgments (Rajalingham et al., 2018). However, a more fine-grained analysis of discrepancies at the level of individual images, typically achieved by comparing confusion matrices, reveals that artificial and biological agents make errors on different images. In comparison to humans, CNNs (i) rely more on texture to classify images, (ii) are more affected by perturbations that degrade image quality, like pixelate noise, spatial frequency filtering or occlusions, and (iii) exhibit robustness and generalizability still lower than biological vision (Ghodrati et al., 2014; Geirhos et al., 2017, 2018; Wichmann et al., 2017; Tang et al., 2018). While we acknowledge that gross similarities in object recognition between artificial and biological neural networks are encouraging, the extent to which existing CNNs reproduce the multiple ways biological agents classify natural images, especially at the level of single items, should not be overstated. These areas of mismatch are important endeavors to steer future research and to improve both neurobiological plausibility and predictive power of CNN models.

### Examining neural correspondence

The overall similarities between CNNs and humans at the behavioral outcomes level motivate comparing their internal processing stages and representational transformations. How well the features learned by CNNs can predict brain responses? To what extent do top-down goals imposed at the output of the CNNs cause hidden layers to respond like real neurons at different stages along the ventral stream hierarchy?

One standard approach is to assess through a regression procedure the correspondence between multi-unit neuronal activity in different ventral stream areas of the primate brain, and the activity of artificial units in different layers of the CNN (Schrimpf et al., 2020). It turns out that neural activity at early stages of the visual hierarchy, like V1, is well predicted by early layers of CNNs that develop Gabor-wavelet-like activation patterns (Cadena et al., 2019). Intermediate areas, like V4, that respond to complex curvature features are best reconstructed from activity in intermediate layers of CNNs, and top hidden layers of CNNs end up being predictive of infero-temporal (IT) neurons (Yamins et al., 2014; Anand et al., 2021). Similar CNN models trained on object categorization also predict responses at early and late stages of the human ventral stream at the aggregate population level of fMRI or MEG data (Cichy et al., 2016; Eickenberg et al., 2017). In this context, Brain-Score is a recent platform to systematically compare different artificial networks for object recognition on how well they approximate the brain’s mechanisms of the ventral stream according to multiple neural and behavioral benchmarks (Schrimpf et al., 2018).

Another popular approach to assess the correspondence between artificial networks and the brain is representational similarity analysis (RSA) (Kriegeskorte, 2008). RSA builds up a distance matrix that represents how dissimilar are the responses for every pair of images presented to an “observer.” Observers can be either biological brains (or specific brain areas) of different species or artificial networks (or their layers). As the dissimilarity is expressed in relative values, it abstracts from the specific methods (neural spikes, fMRI) or the nature of the observer wherein activity is recorded. For example, RSA was used to relate object representation in the IT cortex of humans and monkeys presented with the same images of real-world objects (Kriegeskorte, 2009). The same method has been used to compare different CNNs with representations in the human and monkey IT (Kriegeskorte and Kievit, 2013; Khaligh-Razavi and Kriegeskorte, 2014). These studies showed that better performing CNN models are also more similar to IT, as they develop greater clustering across categories and are also more sensitive to fine-grained dissimilarities within categories (e.g., faces and bodies form subclusters within animate items). In general, it seems that biological and artificial networks both impose upon the visual input certain categorical differentiations that are important for successful behavior.

## Notions of the visual brain commonly overlooked in CNN models

In this section, we outline several notions informing the anatomical and physiological properties of the visual system that still need to be systematically transposed in CNN models through corresponding architectural and computational solutions, respectively. These principles are variably rooted in the process of phylogenetic evolution or acquired from learning in critical developmental periods. They offer insights into the complex interplay between the integration and segregation of functions within the visual system and the constraints that enable the brain to remodel itself through plasticity to compensate for the effects of lesions. Incorporating these notions in CNNs would advance our mechanistic explanation of how complex computations are possible using the machinery available to the biological brains and their driving forces across the life span.

### Multiple routes bypass V1 and target higher-order visual cortices

**FIGURE 1:**
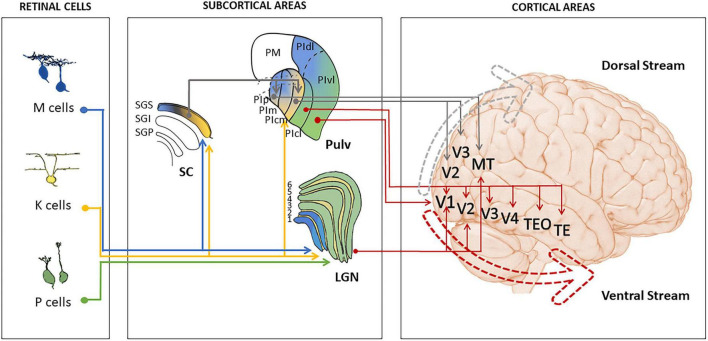
Connections from the retinal ganglion cells to the visual cortex intermediate relays in LGN, SC and Pulv. The blue arrow indicates projections from the M cells in the retina to the superficial layers of SC and magnocellular layers of the LGN. The Yellow arrow indicates the projection from K cells in the retina to the superficial layers of the SC and the intermediate layers of the LGN. The Green arrow indicates projection from the P cells in the retina to the magnocellular layers of the LGN. Gray arrows indicate projections originating from the superior colliculus and reaching the dorsal stream cortical areas via the pulvinar. The red arrows indicate projections from pulvinar subnuclei and LGN to areas along the cortical ventral stream. In LGN and superior colliculus, yellow layers indicate Koniocellular, blue Magnocellular, and green Parvocellular channels. In the pulvinar and SC these pathways are not clearly segregated and shaded blue-yellow; green-blue colors indicate the conjoint presence of the respective channels in given subdivisions. White denotes areas of the superior colliculus and pulvinar not interesting for the present purposes. PIcl, pulvinar inferior centro-lateral; PIcm, pulvinar inferior centro-medial; PIm, pulvinar inferior medial; PIp, pulvinar inferior posterior; PLdm, pulvinar lateral dorso-medial; PLvl, pulvinar lateral ventro-lateral; PM, pulvinar medial; TEO, temporal inferior posterior; TE, temporal inferior anterior.

There are multiple routes through which the visual input reaches the cortex from the retina (Pessoa and Adolphs, 2010; Baldwin and Bourne, 2020). The best-studied route targets V1 after an intermediate relay in the lateral geniculate nucleus of the thalamus (LGN). Standard CNNs loosely model this retino-geniculo-striate pathway with an initial front-end that approximates the retina and the two early layers thereafter. However, multiple pathways bypass V1 and target extra-striate visual areas (including ventral stream areas) through direct and indirect connections from LGN, the pulvinar and the superior colliculus (Bridge et al., 2016; Tamietto and Morrone, 2016; Bruni et al., 2018; McFadyen et al., 2019, 2020). Each of these subcortical structures receives direct projections from the retina. Such projections however, come from different classes of retinal ganglion cells (M, P, and K) specialized to respond to specific visual features (Figure 1).

The characterization of these V1-independent pathways in current CNN models of the visual system is important for several reasons. First, V1-independent pathways are not simply vestigial from a functional and anatomical perspective. After a lesion to V1, extra-striate areas remain responsive from 20% (Schmid et al., 2010) to 80% (Girard et al., 1992) of their pre-lesional activity. The retinal projection to the superior colliculus alone comprises about 100,000 fibers, which is more than the whole human auditory nerve. Second, these alternative pathways contribute to many important functions such as orientation, motion discrimination, object categorization and emotion processing, as these abilities can be retained in patients with V1 damage (Ajina et al., 2015a; de Gelder et al., 2015; Hervais-Adelman et al., 2015; Van den Stock et al., 2015; Ajina and Bridge, 2018; Celeghin et al., 2019). Third, it is becoming increasingly clear that the subcortical structures from which V1-independent pathways originate are not passive relay centers. Instead, they seem endowed with the necessary infrastructure and computational capabilities to instantiate complex analyses of the visual input (Bridge et al., 2016; Georgy et al., 2016; Basso et al., 2021; Carretié et al., 2021; Isa et al., 2021). Lastly, mounting evidence indicates that retino-recipient structures, like the superior colliculus or the pulvinar, provide the developmental foundation of what later in life become complex visual and attentional functions typically ascribed to higher-order cortical areas (Warner et al., 2012; Alves et al., 2022). For example, the superior colliculus has been proposed to establish new-born preferences for faces and facial expressions, and contribute to the maturation of “face patches” in areas of the ventral stream, such as the fusiform gyrus (Johnson, 2005; McFadyen et al., 2020). The pulvinar, through its direct connections to the area middle temporal (MT), drives the early maturation of the dorsal stream, which sustains global motion perception and serves visuo-motor integration (Warner et al., 2015; Kwan et al., 2021).

**FIGURE 2:**
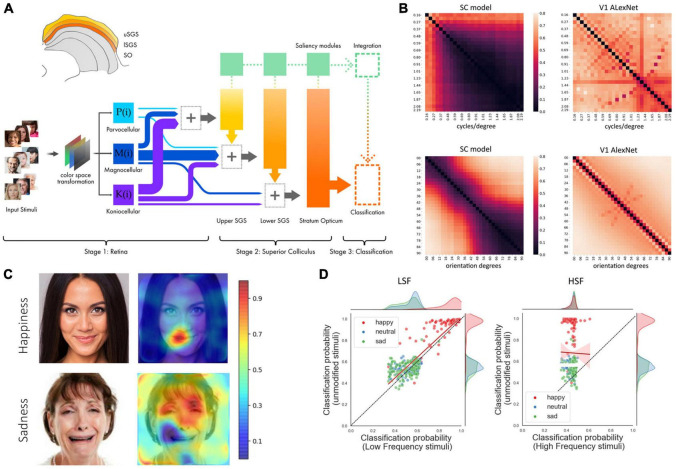
General overview of the model adopted in Méndez et al. (2022) and selected results. **(A)** In the upper left corner, an anatomical diagram of the SC, viewed from a coronal section, highlights the relevant superficial layers that have been modeled with corresponding layers in the CNN. Stage 1: input stimuli are color-transformed and processed. Each one of the three main P, M, and K channels is modeled by a function and the visual information is projected in different proportions to the appropriate SC layers. The varying width of the arrows represent the proportional contribution of each pathway to the corresponding superficial SC layer. Stage 2: the three layers composing the superficial SC are modeled with fractal convolutional blocks and saliency modules that guide attention to select image regions for further analysis. Stage 3: after the final SC layer, the network uses a global averaging stage before a classification layer, while the saliency masks are integrated and contribute to generate the final output of the network. **(B)** Spontaneous emergence of spatial frequency tuning and orientation sensitivity. Discrimination performance across gratings of different spatial frequencies (upper) and orientation (lower) in the SC model and in the AlexNet model that approximate V1 responses. **(C)** Example of bubbles analysis on three subjects from the test dataset. **(D)** Effect of face manipulation toward M and P channels in the SC model and in the fMRI activity of the human SC. Scatterplots and marginal distributions of classification probability for LSF and HSF filtered faces compared to original images. The *y*-axis corresponds to the probability of belonging to the correct category assigned by the model to each unfiltered image, while the *x*-axis represents the probability assigned when the instances are spatial frequency filtered. The red line and shaded area denote the best linear regression fit to the data and its 95% confidence interval. Figure modified from Méndez et al. (2022).

To the best of our knowledge, only one study has built a neurobiologically inspired CNN that simulates the physiological, anatomical, and connectional properties of the retino-collicular circuit and its contribution to facial expression categorization (Méndez et al., 2022; Figure 2). The model consists of a frontend that emulates retinal functions of M, P, and K pathways, along with three layers analogous to the superficial strata of the primate superior colliculus that receive direct retinal information. This CNN matched error patterns and classification accuracy of patients with V1 damage, developed spontaneous tuning to low spatial frequencies in accordance with fMRI data, and generated saliency maps that directed attention to different facial features depending on expressions (Sahraie et al., 2010; Celeghin et al., 2015; Burra et al., 2019). These findings contribute to superseding a cortico-centric perspective on visual functions and to explore with CNNs the encoding of emotional information.

### Dorsal and ventral stream: how many subsystems?

The division of extra-striate visual areas into dorsal and ventral streams is a crucial framework that has been heuristically seminal in visual neuroscience for the past four decades (Ungerleider and Mishkin, 1982; Goodale and Milner, 1992). However, some of its tenets have come under renewed scrutiny (de Haan and Cowey, 2011; Rossetti et al., 2017). For instance, the dorsal pathway is now conceived as a multiplicity of at least three segregate pathways based on different downstream projection targets that serve spatial working memory, visually guided action, and navigation (Kravitz et al., 2011). Similarly, the ventral stream has been proposed to encompass up to six distinct cortico-subcortical systems, each with specialized behavioral, cognitive, or affective functions (Kravitz et al., 2013). More radically, recent evidence suggests the existence of a third visual stream, terminating in the superior temporal sulcus (STS) (Pitcher and Ungerleider, 2021). This third stream appears specialized for the dynamic aspects of social perception and does not fit within the traditional dichotomy altogether.

As described previously, CNN applications have been essentially grounded on models of the ventral stream. However, there are interesting attempts to predict neural responses along the dorsal stream. Using an encoding model, a CNN has been trained to recognize actions in videos and map stimuli to their constituent features (Güçlü and van Gerven, 2017). These features were then regressed to fMRI activity in subjects watching natural movies. Through this method, it was possible to predict responses in the dorsal stream, with deeper layers corresponding to activity in downstream areas such as V3b and MT. Besides a few remarkable exceptions, most studies have generally failed to appreciate that the brain can exploit visual information to achieve different behavioral goals beyond foveal object recognition. These environmental constraints and adaptive pressures shape the functional segregation of different input properties at early encoding stages (Milner and Goodale, 1995). In this context, CNN models can be profitably applied to probe the development of specialized sub-pathways by investigating computational trade-offs and the underlying reasons for the emergence of specialized and segregated sub-systems. For example, a relatively generic architecture can be trained from the same starting point to perform different tasks. Then, the CNN is inspected to understand how many layers can be shared before performance declines and the network needs to split into specialized sub-streams to perform well on all tasks (Kell et al., 2018). Variants of this approach have been recently applied to study why and how face and object processing segregate in the visual system (Rawat and Wang, 2017; Dobs et al., 2019), or to provide computational foundations for dorsal and ventral streams to arise based on different goals and learning principles (Scholte et al., 2018).

### Evaluating hierarchy and linearity of information integration

Uncertainty about how to aggregate the fractioned architecture of the visual brain into pathways also calls into question its hierarchical organization, which also assumes a serial progression of information and linear integration from lower-level to higher-order visual areas. For example, information exchange is reciprocal between adjacent structures, and, in most cases, backward projections outnumber forward projections (Angelucci et al., 2002). Moreover, “shortcut” connections link relatively distant areas: V1 projects directly to V3, V4, and MT; V2 to TEO; and V4 to TE. Developmentally, the traditional view of a hierarchical maturation, where V1 develops first followed by higher-order areas, is contradicted by recent evidence that MT matures in parallel due to driving pulvinar input in early postnatal phases (Bourne and Morrone, 2017). Finally, visual areas like prostriate exhibit response latency, receptive field characteristics, and projection patterns that, to some extent, contradict each other for classifying its hierarchical position and assignment to either the dorsal or ventral stream (Mikellidou et al., 2017; Tamietto and Leopold, 2018).

Concerning hierarchical organization and feed-forward vs. recursive interactions, standard CNNs approximate the initial stages of visual processing (∼150 ms after stimulus onset) when the dominant direction of signal flow within occipito-temporal networks is feedforward (Tang et al., 2018; Semedo et al., 2022). However, rapid feedback interactions coexist with the initial feedforward sweep and can influence basic levels of visual processing, while at longer latencies information exchange gradually reverses to feedback (Bullier, 2001; Semedo et al., 2022). Adding recurrent connections between layers improves equivalence with later stages of neural processing in both the dorsal and ventral stream (Shi et al., 2018; Kar et al., 2019; Kietzmann et al., 2019). One important extension of classical CNNs could thus be to systematically incorporate feedback connections and residual links that skip layers, as they seem to increase receptive field size of corresponding cell units (Jarvers and Neumann, 2019; Rawat and Wang, 2017). In fact, the comparison of different feedback and feedforward architectures suggests that including feedback connections and recurrence at either local and global network level (i.e., within and between layers, respectively) can improve network performance and robustness (Hasani et al., 2019).

## Improving neurobiological plausibility of objective functions and learning rules

As previously mentioned, CNNs are grounded in three essential components: the objective function, the learning rules, and the network architecture (Richards et al., 2019). The principles of the primate visual brain discussed in the previous section can be mainly transposed in the next generation of CNNs through architectural solutions. We now complement the discussion with a focus on approaches aimed at improving the biological plausibility of artificial neural network through objective functions and learning rules.

### Objective functions as ethological task constraints

Animals clearly possess objective functions crucial for survival, which rely variably on both evolution and learning processes. Examples of these functions include escaping predators, recognizing conspecifics, and seeking food. Task constraints are driving forces that shape brain architecture and functions to enhance fitness. Thus, they should be an integral component in developing neurobiologically plausible CNNs. For example, some authors have proposed that the expansion of the visual system, the rise of orbital convergence, and the development of foveal vision evolved to cope with evolutionary pressures favoring the emergence of visually guided reaching and grasping due to the arboreal lifestyle of early primates (Sussman, 1991). These developments have subsequently led to significant improvements in oculomotor behaviors, enabling more efficient visual search and precise localization of targets with minimal head or body movements (Schütz et al., 2011). Other accounts have suggested that detecting snakes before they strike was the primary selective pressure that drove the development of the anthropoids’ visual system, with foveal vision linked to the development of trichromatic color perception (Isbell, 2006).

Recent examples provide insights into the potential benefits of including task constraints as neurobiologically meaningful objective functions in CNN design. Mnih et al. (2014) developed a recurrent CNN that learned to track a simple object without explicit training, reproducing foveation and saccading. Cheung et al. (2017) trained a CNN to perform a visual search task using a retinal front-end with a receptor lattice that could be moved across input images to mimic eye movement, foveating specific features and image parts. The optimization procedure resulted in a virtual retina displaying characteristics of the biological one, featuring high resolution and densely sampled fovea with small virtual receptive fields, and more coarsely sampled periphery with lower resolution and larger receptive fields. Notably, the CNN did not develop these biologically realistic properties when the system was endowed with additional actions, like zooming, which are absent in the biological visual system. These findings suggest that the receptor properties and arrangements in the primate retina can be profitably studied with CNNs, and that several aspects of primate vision may arise from evolutionary pressure to optimize visual world sampling through the integration of eye movements and fixations.

Introducing “visuo-motor” goals as objective functions seems especially ground-breaking when modeling dorsal stream responses that exploit visual information to guide subsequent actions. For example, motion parameters in retinal image must be integrated with oculomotor and vestibular signal to avoid collision, grasp objects, or stabilize gaze on items of interest while we move through the environment (Burr and Morrone, 2022). Accordingly, dorsal stream neuron response properties can be better predicted by a 3D Resnet model trained to orient itself during locomotion (i.e., estimating self-motion parameters from image sequences) compared to networks simply trained on action recognition (Mineault et al., 2021). Therefore, incorporating ethologically relevant tasks as objective functions in CNNs, such as navigation, object manipulation and visual search, can lead to a more comprehensive understanding of the visual brain, and it seems necessary to fully capture the diversity of biological vision, its organizing principles, and relation to other brain functions.

An intriguing research direction in computer vision is neuro-symbolic integration (Kroshchanka et al., 2022), which aims to combine connectionist models with structured knowledge representation. This approach aims to enforce a more structured representation learning by enriching the loss function used to train CNNs with additional domain-distilled information, such as taxonomical relations between objects (Chen et al., 2019; Bertinetto et al., 2020). Visual hierarchies (input images) are matched with ontological hierarchies (enriched loss functions), fostering robustness against adversarial attacks. Examining these models’ behavioral and neural correspondence with the primate visual system presents a promising avenue for research.

### Learning rules and synaptic weights

Learning rules guide the optimization of model parameters, expressed as synaptic weights, to achieve a specific objective function. CNNs typically employ backpropagation, a highly supervised learning process that provides explicit performance feedback (Lindsay, 2021). In contrast, unsupervised learning builds meaningful representations by utilizing the inherent structure of the data (i.e., without instructions). Vision neuroscience has traditionally emphasized unsupervised principles that modulate synaptic changes and local plasticity rules, such as Hebbian learning and spike timing-dependent plasticity (Caporale and Dan, 2008; Chauhan et al., 2021). These principles are embodied in Spiking Neural Networks (SNNs), where network units process time-varying spikes at the input and output stages, mimicking time-dependent processing in natural vision (Maass, 1997; Tavanaei et al., 2019).

Backpropagation calculates gradients and adjusts weights between nodes during learning, relying on biologically unrealistic assumptions, such as symmetrical feedback weights and separate forward and backward information flows (McClelland et al., 1986; Bengio et al., 2017). Despite this, biological brains can approximate backpropagation learning when assumptions about inhibitory microcircuits, short-term plasticity, or feedback connections are considered (Körding and König, 2001; Roelfsema and Van Ooyen, 2005; Lillicrap et al., 2016; Guerguiev et al., 2017; Scellier and Bengio, 2017; Whittington and Bogacz, 2017; Pozzi et al., 2018; Sacramento et al., 2018). Dynamic weight sharing has been recently proposed as a learning rule capable of accounting for local weight updates. It facilitates local weight adjustments via lateral connections, enabling local neuron subgroups to equalize weights through shared activity and anti-Hebbian learning (Pogodin et al., 2021). Artificial networks with dynamic weight sharing exhibit a better fit to ventral stream data, as measured by the Brain-Score, performing almost as well as traditional CNNs.

Recent studies on synaptic plasticity and learning have focused on top-down attentional mechanisms and predictive coding, both involving feedback connectivity and predicting varied activity distributions (Kwag and Paulsen, 2009; Yagishita et al., 2014; Bittner et al., 2017; Lacefield et al., 2019; Williams and Holtmaat, 2019). For instance, the influence of fronto-parietal attentional network over the visual system has been traditionally modeled in CNNs using saliency modules that guide visual selection for further processing of the most informative image parts (Itti et al., 1998). Additionally, attentional learning modules have been adapted to encode a topographic saliency map of the visual scene generated by the superior colliculus (Méndez et al., 2022).

Non-invasive imaging techniques, such as fMRI and wide-field calcium imaging, enable measuring the dynamics of representational changes and comparing learning trajectories during training. Estimating synaptic changes *in vivo* and relating them to behavioral performance can facilitate comparisons between artificial and biological brains based on learning procedures, rather than solely on the final representations they generate.

## Toward causal evidence: closed-loop experiments and task constraints

### Closed-loop experiments through image synthesis

Successful application of AI in neuroscience should permit moving the research agenda beyond correlations toward new approaches that gather causal evidence of the predictive link between artificial and biological brains. “Closed-loop” experiments harness CNNs activations to systematically manipulate brain activity in pre-defined visual regions of the brain, such as V1 and V4, according to the following logic (Bashivan et al., 2019; Ponce et al., 2019). First, a CNN presented with natural images is trained to predict neural activity recorded in the real brain, wherein the same images are shown to the animal. Then, the CNN is used to synthesize optimal images that maximally excite specific artificial units (or layers) by selecting their preferred features. Finally, when these synthetic images are shown to the real neurons, their responses are measured and found to match the predicted firing rate. This demonstrates that the CNN can capture the correspondence from pixels to neural responses (Olah et al., 2017; Walker et al., 2019). By enabling non-invasive control over brain activations, this closed-loop approach promises new causal insights into the interplay of multiple brain areas during visual processing. For example, the method permits stringent control of activity in one brain region while establishing the impact on the functioning of another related area.

### Lesion analysis at the single neuron and population level

Lesion-symptom mapping is probably the most straightforward tool in neuroscience to establish the causal contribution of a neural structure to a given function and to investigate the plastic changes that intervene thereafter. However, this approach has inevitable limitations when applied to biological brains. Performing single-neuron ablations has traditionally proved challenging due to technical limitations (Wurtz, 2015). The advent of optogenetics, viral vectors, and two-photon stimulation techniques promise to overcome these challenges (Kinoshita and Isa, 2015; Kinoshita et al., 2019; Vanduffel and Li, 2020; Klink et al., 2021). However, these methods are still in infancy and their application to animal models phylogenetically proximal to humans has just begun. In primates, surgical lesions are still the most used approach at the areal or network level of analysis. Nevertheless, their precision and specificity vary depending on multiple factors, whereas, in humans, naturally occurring lesions obviously do not adhere to cytoarchitectonic or functional boundaries between areas.

Artificial networks can fulfill “*in silico* neurophysiology” at the single cell level exceedingly well, as we can characterize every unit’s activity in response to predefined ablation and measure the impact on neural computation and performance (Barrett et al., 2019). This approach revealed that CNN accuracy drops as increasing numbers of neurons are deleted (Morcos et al., 2018). Moreover, networks that learn generalizable solutions are more robust to ablations than those that simply memories the training data (Zhou et al., 2018). Notably, neurons with clearly defined tuning properties are not more important for classification performance than those with complex or ambiguous tuning properties, as the latter often contains substantial amounts of task-relevant information. These findings contribute to reconsidering some basic assumptions in neurophysiology, where single-cell selectivity to stimulus features or categories has been traditionally regarded as the principal proxy to infer functions.

Drop-out, a randomized temporal ablation technique, is widely used in training artificial neural networks to ensure robustness. Although predominantly employed for regularization, it can also profitably simulate “virtual lesions” and the resulting plasticity. To model neuroplasticity using CNNs, researchers can simulate the reorganization of neural connections and the emergence of new response properties following lesions by adjusting the network’s architecture, connectivity, or learning rules. Neuroplasticity can be measured by evaluating the network’s adaptability to external perturbations, such as introducing noise or limiting weight updates in a specific layer. This approach may reveal how mid-level features and new response properties emerge as the network compensates for the loss of specific neural elements. For example, how do response properties in intact visual structures change following brain damage? Neural tuning in extra-striate visual areas gradually recovers after V1 damage. However, this recovery does not lead extra-striate neurons to emulate the response properties of the damaged cortex but to resume their own original response properties (Maffei and Fiorentini, 1973; Guido et al., 1992). Furthermore, in humans, if V1 damage occurs in adulthood, the response in MT neurons for motion and contrast reshapes to resemble the response pattern of V1 in the intact brain (Ajina et al., 2015b).

Lesion analysis can be applied to deep learning architectures incorporating features like lateral connection or shortcut and learning through an online procedure, where weights are updated one data sample at a time. Investigating how shortcut connections serve as alternative information pathways could also yield valuable insights. CNNs can be exploited to address these issues with new tools offering insights into the mechanisms underlying neuroplasticity and potentially guiding the development of interventions to promote recovery after brain damage.

## Conclusion

The resurgence of interest in neural networks has sparked both enthusiasm and skepticism regarding the relevance of CNNs in understanding biological brains (Michel et al., 2019). Experimenting with CNNs offers valuable insights for neuroscience, especially if biological credibility is recognized as a crucial factor in modeling network properties, and results are deployed in assays on biological brains. In turn, laboratory investigations should drive the design of future CNN models. This iterative process offers a principled perspective to specifying mechanistic hypotheses on how real brains may carry out visual and cognitive functions.

Longstanding questions include whether perceptual representations, like sensitivity to biological motion or face recognition, are innate or learned from experience (Behrmann and Avidan, 2022). While traditional supervised models used for explaining primate object recognition demand vast labeled data, primates develop sophisticated object understanding with limited training and less examples (Saxe et al., 2020). However, (quasi)innate behaviors can partly be conceived as learned on an evolutionary timescale, and the relationship between evolutionary and developmental variations can be reframed in CNNs. Evolutionary diversity can be addressed by changing architectural parameters that restructure the computational primitives of the network, while development can be modeled by modifying filter parameters and their learning algorithms, imitating synaptic weights.

To align CNNs with biology and to steer future directions, it seems useful to consider neural responses and their functions as an emerging consequence of the interplay between objective functions, learning rules and architecture. The environment and its constraints seem to provide guidance in identifying which objective functions are useful for biological brains to optimize. Accordingly, introducing ethologically relevant tasks as objective functions in CNNs, such as navigation, object manipulation, and visual search, can lead to a more comprehensive understanding of the visual brain.

By exploring unsupervised learning principles and spiking neural networks, researchers can better understand the role of local plasticity rules and time-varying signals in natural vision, thereby improving the neural correspondence of CNNs. This may involve exploring alternative learning algorithms, such as reinforcement learning, that incorporate elements of reward-based learning and decision-making. Analyzing the processing of affective signals also offers a testing ground for the proposal that representation formation is driven by the need to predict the motivational value of experience and its interface with attention (Mnih et al., 2015).

Architecturally, it is important to incorporate the role of subcortical structures and V1-independent pathways in visual processing. This would quantify the respective contributions of redundancy and synergy in the multiplicity of parallel routes that help decode visual stimuli (Nigam et al., 2019; Luppi et al., 2022). Systematically integrating feedback connections, recurrent connectivity motifs, and residual links can enhance performance, robustness, and equivalence with the brain’s hierarchical organization and the balance between linear and recursive interactions. CNN models can be utilized to probe the development of functional segregation and the emergence of specialized subsystems through computational trade-offs.

The use of closed-loop experiments and lesion analysis can provide new causal insights into the predictive link between artificial and biological brains, the mechanisms underlying neuroplasticity, and the development of interventions to promote recovery after brain damage. For example, what is the impact of silencing a single structure on the computations performed in other parts of the network at both the aggregate level of layers and of single units? In CNN, this would imply assessing network robustness to “virtual ablations” of individual components and can help evaluate how the biological brain recruits plasticity.

Convolutional Neural Networks face the unavoidable trade-off between complexity, interpretability, and energy consumption (Petri et al., 2021). On this front, the sparsity of spike computing is central to information processing where computationally demanding tasks can be realized by a restricted subset of neurons and disentangled from millions of examples through “direct fit” (Olshausen and Field, 2004; Dalgleish et al., 2020; Hasson et al., 2020). Transformer models originally applied to natural language tasks are finding their way in the vision science community (Khan et al., 2022). Unlike CNNs, transformers support parallel processing, require minimal inductive biases for their design, and allow simultaneous processing of multiple modalities.

The development of CNNs is progressing rapidly and spreading in different directions and domains within neuroscience. The theoretical discussion and sober consideration of the promises and pitfalls accompanying these developments are needed to ensure that neuroscientists make informed use of CNNs as falsifiable models of biological brains. By addressing these critical areas, researchers can harness the full potential of CNNs to bridge the gap between neuroscience and cognitive sciences, ultimately leading to a deeper understanding of the primate visual system and advancing artificial intelligence.

## Author contributions

AC, CAMG, and MT contributed to the conception of the study. MT, AC, CAMG, and AB wrote the first draft of the manuscript. DO, MD, AP, and GP wrote sections of the manuscript. All authors contributed to manuscript revision, read, and approved the submitted version.
